# Placenta-derived IL-32β activates neutrophils to promote preeclampsia development

**DOI:** 10.1038/s41423-021-00636-5

**Published:** 2021-03-11

**Authors:** Dan Liu, Qiang Li, Hailin Ding, Guangfeng Zhao, Zhiyin Wang, Chenrui Cao, Yimin Dai, Mingming Zheng, Xiangyu Zhu, Qianwen Wu, Ya Wang, Honglei Duan, Huirong Tang, Xianyan Lu, Yayi Hou, Yali Hu

**Affiliations:** 1 Department of Obstetrics and Gynecology, The Affiliated Drum Tower Hospital of Nanjing University Medical School, Nanjing, China; 2 Department of Obstetrics and Gynecology, Drum Tower Clinic Medical College of Nanjing Medical University, Nanjing, Jiangsu China; 3 The State Key Laboratory of Pharmaceutical Biotechnology, Division of Immunology, Medical School, Nanjing University, Nanjing, China

**Keywords:** IL-32, Neutrophil, Preeclampsia, ROS, HUVEC, Cytokines, Innate immune cells

## Abstract

Immune activation at the maternal-fetal interface is a main pathogenic factor of preeclampsia (PE). Neutrophils (PMNs) are activated in PE patients, but the mechanism and consequences of PMN activation need to be further explored. Here, we demonstrated that interleukin-32 (IL-32) expression was significantly upregulated in syncytiotrophoblasts (STBs) and that IL-32β was the major isoform with increased expression in the placenta of severe PE (sPE) patients. Furthermore, the level of IL-32 expression in the placenta was correlated with its level in the serum of sPE patients, indicating that IL-32 in the serum is derived mainly from the placenta. Then, in vitro experiments showed that IL-32β could highly activate PMNs and that these IL-32β-activated PMNs were better able to adhere to endothelial cells (HUVECs) and enhance the expression of vascular cell adhesion molecule-1 (VCAM-1) and intercellular cell adhesion molecule-1 (ICAM-1) in HUVECs, which could be reversed by preincubation with the NADPH oxidase inhibitor VAS 2870. In addition, we showed that IL-32β mainly activated PMNs by binding to proteinase 3. Finally, IL-32β administration induced a PE-like phenotype in a pregnant mouse model. This study provides evidence of the involvement of IL-32β in the pathogenesis of PE.

## Introduction

Preeclampsia (PE) is a serious complication in pregnancy that threatens the health and even the life of the mother and fetus^1^. Although the mechanisms underlying PE remain elusive, disturbance of maternal immune tolerance to the semiallogeneic fetus is recognized as one of the key pathologies of PE^2–5^. There are two distinct immunological interfaces in pregnancy: The first is between maternal immune cells in the decidua and the fetal trophoblast and dominates during early pregnancy, and the second involves interactions between circulating maternal immune cells and syncytiotrophoblasts (STBs) and becomes the dominant interface towards the end of pregnancy, which is considered to indicate that placenta-derived circulating factors activate maternal immune cells and induce systemic endothelial dysfunction^4,6,7^.

Neutrophils (PMNs) are the most abundant immune cells in the peripheral blood, and it has been reported that these cells are markedly activated in PE^8^. PMNs in PE pregnancy produce a large number of reactive oxygen species (ROS)^9,10^ and express high levels of the adhesion molecules CD11b and intercellular cell adhesion molecule-1 (ICAM-1)^11–13^, which may increase the number of PMNs adhered to the endothelium and infiltrated in the intimal space of the maternal systemic vasculature^11,12^. Some studies have shown that the placental villus culture supernatant^14^ and plasma^15^ of PE patients stimulate activation of maternal PMNs. Thus, PMNs may play a vital role in communicating between the preeclamptic placenta and maternal vascular endothelium^9,10,16^. However, the mechanism underlying PMN activation in PE is still unclear.

Interleukin-32 (IL-32) is an inflammatory cytokine that is widely expressed in various human tissues and organs^17^ and regulates cell growth, metabolism and immunity; IL-32 is therefore involved in several inflammatory diseases^18–24^. IL-32 has nine alternatively spliced isoforms including IL-32α, IL-32β, IL-32γ, IL-32δ, IL-32ε, IL-32ζ, IL-32η, IL-32θ, and IL-32 small (sm), all of which show differences in terms of effects and potency in eliciting a specific effect^25^. The specific receptor for IL-32 has not yet been identified. However, it has been reported that proteinase 3 (PR3) may be a binding receptor of IL-32^26,27^. In addition, IL-32 may also bind to the cell-surface integrins α_V_β_3_ and α_V_β_6_^28,29^. To date, whether IL-32 is expressed in the placenta and whether it contributes to the activation of PMNs in PE patients are still unknown.

In the present study, we demonstrated that IL-32 expression was significantly upregulated in the STBs and serum of severe PE (sPE) patients compared with gestational age-matched normal controls. IL-32β was the major isoform exhibiting increased expression in the placenta and could highly activate PMNs. Pretreatment of PMNs with IL-32β increased the adhesion of PMNs to vascular endothelial cells, promoting expression of ICAM-1 and vascular cell adhesion molecule-1 (VCAM-1) in the endothelial cells. The effect of IL-32β on PMNs could be inhibited by an NADPH oxidase (NOX) inhibitor and might be mediated though binding to PR3. Importantly, intravenous administration of IL-32β induced a phenotype of PE (hypertension, proteinuria, and fetal growth restriction) in a pregnant mouse model.

## Materials and methods

### Placenta and serum sample collection

This study was reviewed and approved by the Ethics Committee of Drum Tower Hospital, Nanjing University Medical School. Written informed consent was obtained from all participants. The placental tissue samples and serum samples used in this study were obtained from pregnant women at the Department of Obstetrics and Gynecology of Drum Tower Hospital from November 2016 to June 2020 and stored in the Jiangsu Biobank of Clinical Resources. Placental tissue samples collected from 12 normal pregnant women who experienced noninfected preterm birth (nPTB) and 22 sPE patients were used for western blotting, immunostaining and PCR. The clinical characteristics of the two groups are summarized in Table 1. Serum samples collected from 27 normal pregnant women and 36 sPE patients were used to detect the secreted IL-32 level. The clinical characteristics of the pregnant women who provided serum are summarized in Table 2.

**Table 1:** Clinical characteristics of pregnant women collected for placentas (values are mean ± SD)

Analyzed item	nPTB, *n* = 12	sPE, *n* = 22	*P* value
Maternal age, years	28.50 ± 3.83	29.73 ± 5.14	>0.05
Gestational age, weeks	35.11 ± 3.79	34.66 ± 3.96	>0.05
BMI, kg/m^2^	22.68 ± 3.29	24.00 ± 2.68	>0.05
Systolic BP, mmHg	117.6 ± 11.31	167.4 ± 12.23	<0.05
Diastolic BP, mmHg	69.92 ± 6.05	103.5 ± 11.05	<0.05
Proteinuria	–	+ to + +++	<0.05
Fetal weight, g	2680.00 ± 680.1	2133.00 ± 942.40	>0.05
Placental weight, g	480.00 ± 117.00	412.50 ± 116.30	>0.05

*BP* blood pressure, *nPTB* gestational age-matched noninfected preterm birth; BMI at 20 weeks of gestation

**Table 2:** Clinical characteristics of pregnant women collected for sera (values are mean ± SD)

Analyzed item	NP, *n* = 27	sPE, *n* = 36	*P* value
Maternal age, years	30.15 ± 4.21	30.58 ± 5.21	>0.05
Gestational age, weeks	33.57 ± 2.76	34.63 ± 3.79	>0.05
BMI, kg/m^2^	23.76 ± 3.046	24.32 ± 2.60	>0.05
Systolic BP, mmHg	115.70 ± 8.76	166.90 ± 15.59	<0.05
Diastolic BP, mmHg	68.11 ± 7.46	102.90 ± 13.28	<0.05
Proteinuria	–	+ to + +++	<0.05

*BP* blood pressure, *NP* normal pregnancy; BMI at 20 weeks of gestation

### Human PMN isolation

Human PMNs were isolated from the heparin anticoagulant-treated peripheral blood of healthy pregnant donors following a protocol reported previously^30^. Briefly, erythrocytes were sedimented by adding a quarter volume of a dextran/saline solution (6% dextran T-500 in 0.9% NaCl) at room temperature for 20 min. The erythrocyte-depleted supernatants were then layered on Lymphoprep (1.077± 0.001 g·mL^−1^, Axis-Shield PoC AS, Oslo, Norway) and centrifuged at 2000 rpm and room temperature for 20 min. Contaminating erythrocytes in the PMN pellets were lysed by treatment with eBioscience 1X RBC Lysis Buffer (REF: 00–4333, eBioscience, San Diego, CA, USA) for 10 min at 4 °C. PMNs were then resuspended in RPMI 1640 medium (Gibco/Invitrogen, Carlsbad, CA, USA) containing 10% heat-inactivated FBS (Gibco/Invitrogen) at a density of 1 × 10^6^ cells per mL and maintained at 37 °C in a humidified atmosphere with 5% CO_2_. PMN purity was >98%, as determined by both Wright-Giemsa staining and FACS analysis with anti-CD14-FITC (clone: 61D3, eBioscience) and anti-CD66b-APC (clone: G10F5, eBioscience) antibodies.

### HUVEC isolation

Primary HUVECs were isolated from the umbilical vein vascular wall by 0.5 mg·mL^−1^ collagenase (C0130, Sigma-Aldrich, St. Louis, MO, USA) treatment for 13 min at 37 °C. The cells were then cultured in RPMI 1640 medium containing 20% FBS, 5 μg·mL^−1^ endothelial cell growth supplement (E2759, Sigma-Aldrich) and antibiotics (100 U·mL^−1^ penicillin and 100 μg·mL^−1^ streptomycin) at 37 °C in a humidified incubator containing 5% CO_2_.

### Immunostaining

Placental tissues were collected from the maternal surface of placentas near the root of the umbilical cord and fixed with 4% paraformaldehyde overnight. Then, the tissues were embedded in paraffin and sectioned at a thickness of 2 μm. After deparaffinization, endogenous peroxidase activity was blocked with 3% H_2_O_2_. Slides were pretreated using heat‐mediated antigen retrieval with sodium citrate buffer or Tris/EDTA buffer for immunohistochemistry (Typing, Nanjing, Jiangsu, China). The sections were incubated with primary antibodies against IL-32 (1:150, Ab37158, Abcam, Cambridge, MA, USA), CK7 (1:500, Ab181598, Abcam), and Neutrophil Elastase (1:200, Ab68672, Abcam) at 4 °C overnight. Negative controls were performed by substituting the primary antibodies with the same concentration of preimmune rabbit or mouse IgG or by omitting the primary antibodies. After incubation with HRP‐conjugated secondary antibodies, the sections were exposed to DAB to visualize the antigen signals and counterstained with hematoxylin. The sections were viewed and imaged under a microscope (DM6 B, Leica, Wetzlar, Germany).

Frozen placental sections were fixed using 4% paraformaldehyde in PBS at pH 7.4 for 10 min at room temperature. After three washes with PBS, the slides were blocked in 2% BSA in PBST (PBS + 0.1% Tween-20) for 1 h and then incubated with diluted primary antibodies for 4 h in a humidified chamber at RT. Positive signals were visualized by incubation with fluorophore-conjugated secondary antibodies (Jackson ImmunoResearch, West Grove, PA, USA). The slides were mounted with mounting medium containing DAPI (Abcam) and then viewed and imaged under a fluorescence microscope (DM6 B, Leica). Antibodies against CK7 (1:500, Ab181598, Abcam) and CD66b (1:20, G10F5, eBioscience) were used for immunofluorescence.

### Reverse-transcription PCR and quantitative real-time PCR analysis

Total RNA was extracted from cells or tissues with TRIzol Reagent (Invitrogen, Carlsbad, CA, USA) according to the manufacturer’s instructions. RNA samples were treated with DNase I (Promega, San Luis Obispo, CA, USA) to remove any contaminating genomic DNA and then reverse transcribed into cDNA with random primers in a 20-μL reaction. Quantitative real-time PCR (qPCR) assays were performed on a Light Cycler 480 II detection system (Roche, Pleasanton, CA, US) using SYBR Green PCR Master Mix. Relative gene expression levels were calculated with the 2^(-ΔCT)^ method and normalized to the level of GAPDH. The primers used in this study are listed in Table S1.

### Transcriptome analysis

PMNs were isolated from the peripheral blood of three normal pregnant women and treated with 100 ng·mL^−1^ IL-32β for 4 h. Control PMNs were treated with the same volume of PBS. Total RNA was extracted using TRIzol reagent. RNA quality was assessed on an Agilent 2100 Bioanalyzer (Agilent Technologies, Palo Alto, CA, USA) and checked using RNase-free agarose gel electrophoresis. Transcriptome sequencing analysis was performed on an Illumina HiSeq2500 by Gene Denovo Biotechnology Co. (Guangzhou, China). Differential expression analysis between two different groups was performed with DESeq2 software. Genes with a false discovery rate (FDR) below 0.05 and absolute fold change≥2 were considered differentially expressed genes (DEGs). Gene Ontology (GO) biological process enrichment analysis and KEGG pathway enrichment analysis were performed for all DEGs. Gene set enrichment analysis (GSEA) was performed to analyze ranked lists of all available genes.

### Western blot analysis

Placental tissues were lysed in lysis buffer (Biosharp, Hefei, Anhui, China) supplemented with a protease inhibitor cocktail (MedChemExpress, Monmouth Junction, NJ, USA). The protein concentration was determined using the Pierce BCA Protein Assay Kit (REF: 23225, Thermo Fisher Scientific, Waltheam, MA, USA). Protein lysates (40 µg) were loaded onto a 10% SDS‐PAGE gel and then transferred to a PVDF membrane (Millipore, Billerica, MA, USA). The membrane was labeled with primary antibodies against IL-32 (1:100, ab37158, Abcam) overnight at 4 °C and then incubated with a horseradish peroxidase (HRP)‐conjugated secondary antibody (1:1000, 7074 S, Cell Signaling Technology, Danvers, MA, USA) for 2 h at room temperature. GAPDH (1:2000, AC035, Abclonal, Wuhan, Hubei, China) was used as an internal standard. The signals were visualized with an ECL solution (Millipore). Images were obtained using a MiniChemiTM Chemiluminescence imaging system (Sage Creation, Beijing, China).

### Flow cytometry

To measure the levels of ROS, PMNs (1*10^6^ cells·mL^−1^) were treated with different concentrations of recombinant IL-32β (6769-IL-025, R&D Systems, Minneapolis, MN, USA) for 2 h, 4 h or 8 h. Then, the PMNs were collected and incubated with DCFH-DA (1:1000, S0033, Beyotime, Shanghai, China) in RPMI 1640 medium for 20 min at 37 °C according to the manufacturer’s instructions. After washing with RPMI 1640 medium three times, the samples were analyzed by flow cytometry. To evaluate the levels of CD11b and CD66b, PMNs treated with or without IL-32β for 1, 2 or 4 h were labeled with anti-CD11b-APC (clone: ICRF44, eBioscience) or anti-CD66b-APC (clone: G10F5, eBioscience) antibodies for 30 min at 4 °C. All isotype-matched controls were purchased from eBioscience. The apoptosis of PMNs treated with or without IL-32β for 8 h was detected using an Annexin V-Alexa Fluor 647/PI apoptosis kit (FMSAV647-100, FcMACS, Nanjing, China). In several experiments, PMNs were preincubated with VAS 2870 (6654, Tocris, Ellisville, MO, USA), α-1-antitrypsin (SRP6312, Sigma-Aldrich) or Echistation, α1 isoform (3202, Tocris) for 1 h before IL-32β treatment. Flow cytometry was performed on an Accuri C6 flow cytometer (Becton Dickinson, Franklin Lakes, NJ, USA) or a CytoFLEX flow cytometer (Beckman Coulter, Fullerton, CA, USA). Data were analyzed using FlowJo software (TreeStar, San Carlos, CA, USA) or CytExpert 2.3 (Beckman Coulter).

### Wright-Giemsa staining

Fresh or pretreated PMN smears were stained with a modified Wright-Giemsa stain (Baso Diagnostics, Zhuhai, Guangdong, China) for 30 s. Then, a double volume of 1× PBS was added and incubated for 3 min. After washing with water, the slides were dehydrated and mounted with resinous mounting medium.

### Phagocytosis assay

PMNs (1*10^6^ cells·mL^−1^) were pretreated with or without 100 ng·mL^−1^ IL-32β for 4 h and plated in a 96-well plate at 1*10^6^ cells/well in 100 µL of pHrodo zymosan suspension (0.5 mg·mL^−1^, P35364, Invitrogen) per well. The plate was incubated for 1 h at 37 °C to allow phagocytosis and acidification. For negative controls, PMNs were incubated in 100 µL of pHrodo zymosan suspension for 1 h at 4 °C. Then, the PMNs were collected and analyzed by flow cytometry with an Accuri C6 flow cytometer. Data were analyzed using FlowJo software.

### PMN adhesion assay

Purified human PMNs were labeled with calcein-AM (2 µg·mL^−1^, C3099, Invitrogen) for 15 min at 37 °C and then washed twice with PBS. Then, the cells were plated in a 12-well plate at 1*10^6^ cells·mL^−1^ and treated with or without 100 ng·mL^−1^ IL-32β for 1 h. The pretreated PMNs were collected and washed twice with PBS. The cells were applied to a monolayer of HUVECs in a 24-well plate at 2*10^5^ cells/well and incubated for 1 h at 37 °C. Next, any nonadherent cells were removed by gently washing the wells twice with PBS. The number of adherent PMNs was counted under a microscope (DMi8, Leica) and averaged from at least eight randomly selected fields of view per well. To study the effect of ROS, PMNs were preincubated with 10 µM VAS 2870 for 1 h before IL-32β treatment.

### PMN and HUVEC coculture

Freshly isolated PMNs were seeded at 1*10^6^ cells·mL^−1^ in a 24-well plate and treated with or without 100 ng·mL^−1^ IL-32β for 1 h at 37 °C in a humidified incubator containing 5% CO_2_. Then, the pretreated PMNs were collected and washed with PBS twice. The cells were applied to a monolayer of HUVECs in a 24-well plate at 1*10^6^ cells·mL^−1^. After incubation for 24 h at 37 °C, the culture medium was collected to detect sICAM-1 and VCAM-1. VAS 2870 was added to the culture medium 1 h before IL-32β treatment to study the effect of ROS.

### Cytokine measurements

Serum samples were collected from patients with sPE or a gestational age-matched normal pregnancy to detect the secreted IL-32 level using an enzyme-linked immunosorbent assay (ELISA) kit purchased from Sino Biological (IL-32 ELISA Kit, Human; Cat: KIT11064). The levels of IL-1β (DLB50, R&D Systems), TNFα (DTA00D, R&D Systems), sICAM-1 (ELH-ICAM1, RayBiotech, Peachtree Corners, GA, USA) and VCAM-1 (ELH-VCAM1, RayBiotech) in culture medium were measured with ELISA kits and conducted according to the standard procedure.

### Animal model

C57BL/6 J female and male mice were purchased from Beijing Vital River Laboratory Animal Technology Co., Ltd. The female mice were 10–12 weeks old and weighed 20–25 g. The male mice were 12–20 weeks old and weighed 25–30 g. Animals were housed in a pathogen-free, temperature- and humidity-regulated environment on a 12-h light cycle. Pregnancy was achieved by housing female and male mice together from 8 p.m. to 8 a.m. the next morning. Gestational day 0.5 (GD0.5) was determined by the presence of vaginal spermatozoa. Pregnant mice were randomly divided into three groups for injection of saline (*n* = 7), recombinant IL-32β-1 μg (*n* = 7) or IL-32β-3 μg (*n* = 7) via the tail vein on GD8.5, GD11.5, GD14.5, and GD16.5. The dosage of IL-32β was determined from the literature^31–33^. Blood pressure and proteinuria were measured as described in a previous study^34^. Blood pressure was monitored by tail-cuff plethysmography (BP-2010A, Softron, Beijing, China) on GD0.5, GD5.5, GD12.5 and GD15.5. Urine (6:00 p.m.–8:00 a.m.) was collected from fasted dams housed individually in metabolic cages on GD0.5 and GD13.5 to measure proteinuria, which was calculated as urinary protein = proteinuria concentration × urine volume. Pregnant mice were sacrificed to collect peripheral blood, fetuses and placentas on GD17.5. To detect PMNs, fresh blood was labeled with anti-CD45-APC-Cy7 (clone: 30-F11, BD Biosciences, Franklin Lakes, NJ, USA), anti-CD11b-FITC (clone: M1/70, BD Biosciences) and anti-Ly6G-APC (clone: 1A8, BD Biosciences) antibodies for 30 min at 4 °C. Erythrocytes were removed by treatment with eBioscience 1X RBC Lysis Buffer for 5 min at RT. All procedures were approved by the Ethics Review Board for Animal Studies of Drum Tower Hospital.

### Statistical analysis

Data analysis was performed using GraphPad Prism (GraphPad Software, San Diego, CA, USA). Differences between two groups were analyzed by Student’s *t* tests. Multigroup comparisons were performed by one-way ANOVA or two-way ANOVA. Data are presented as the mean ± SD. For all statistical tests, a value of *P* < 0.05 was considered to be significantly different.

## Results

### The level of IL-32 in the placenta is upregulated and correlated with the serum level in sPE patients

**Fig. 1:**
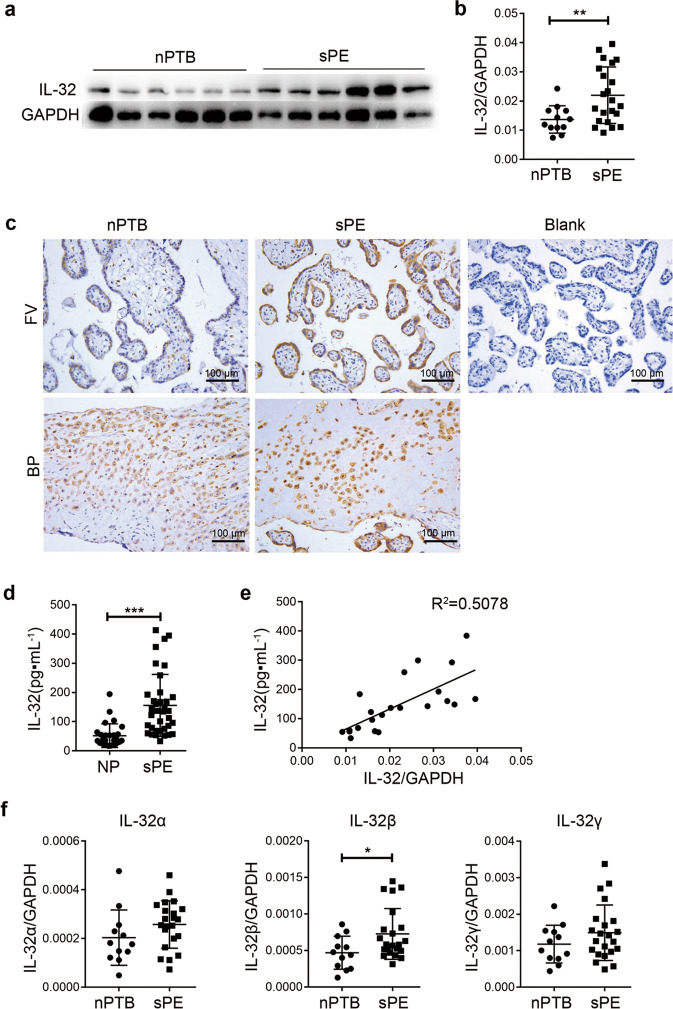
IL-32 levels are upregulated in the placenta and serum of patients with sPE. **a** Immunoblotting for IL-32 in placental lysates from patients with sPE (*n* = 6) or nPTB (*n* = 6). **b** q-PCR analysis of IL-32 in placental tissues from patients with sPE (*n* = 22) or nPTB (*n* = 12). **c** Immunostaining for IL-32 on the free villous (FV) and BP of placental sections from patients with sPE (*n* = 8) or nPTB (*n* = 8). Scale bar = 100 μm. **d** ELISA analysis of the IL-32 concentration in the serum of sPE patients (*n* = 36) and gestational age-matched NP (*n* = 27) women. **e** The correlation of the IL-32 concentration in serum samples and IL-32 expression level in placentas (*n* = 22) from sPE patients. **f** q-PCR analysis of IL-32 isoforms such as IL-32α, IL-32β, and IL-32γ in placental tissues from patients with sPE (*n* = 22) or nPTB (*n* = 12). Error bars, mean ± SD. The data were analyzed by an unpaired *t* test. *P < 0.05; **P < 0.01; ***P < 0.001

To study the role of IL-32 in PE, we evaluated IL-32 expression in human placenta tissue from patients with sPE (*n* = 22) and nPTB (*n* = 12) (Fig. 1). Compared with controls, sPE patients showed significantly upregulated IL-32 expression in the placenta (Fig. 1a, b), as detected by both western blotting and q-PCR. Immunohistochemistry results showed that IL-32 expression was mainly upregulated on STBs of sPE patients, while it was only slightly increased on cells in the basal plate (BP) (Fig. 1c). As shown in Fig. S1, in the placental tissues of normal pregnant women at different gestational stages, IL-32 was strongly expressed on STBs in first-trimester (TM) placentas and progressively downregulated in second- and third-TM placentas (Fig. S1a). In addition to STBs, villous cytotrophoblasts (CTBs), column CTBs and blood vascular endothelial cells also expressed IL-32 in the first- and second-TM placentas, but the intensity was much lower than that on STBs (Fig. S1). In the third-TM placentas, the expression levels of IL-32 were low on STBs, Extravillous trophoblast cells (EVTs) and some other cells (Fig. S1).

Next, the levels of IL-32 in serum samples from 36 sPE patients and 27 gestational age-matched NP women were detected by ELISA. The results showed that serum IL-32 levels were significantly increased in the patients with sPE (Fig. 1d, NP vs sPE: 51.98 ± 40.56 vs 155.8 ± 106.08 pg·mL^−1^). Furthermore, we analyzed the correlation between the expression of IL-32 in the placenta and the level of IL-32 in the serum of sPE patients (*n* = 22). The results showed that the IL-32 level in the serum was positively correlated with the IL-32 expression in the placenta of sPE patients (*R*^2^ = 0.5078) (Fig. 1e). Since IL-32 has alternatively spliced isoforms, specific primers were designed to detect the three major isoforms, IL-32α, IL-32β, and IL-32γ. We identified that IL-32β expression but not that of the other major isoforms was significantly increased in the placenta of sPE patients (Fig. 1f).

### PMN levels are increased in both the peripheral blood and placenta of sPE patients

**Fig. 2:**
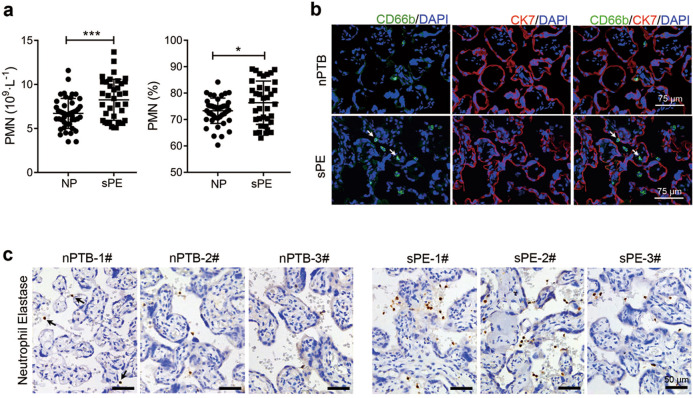
The PMN level is increased in the peripheral blood and placenta of sPE patients. **a** The number and percentage of PMNs in the peripheral blood of women with sPE (*n* = 37) or NP (*n* = 46). **b** Immunofluorescence staining for CD66b, which is a marker of PMNs, and CK7 in placental sections from patients with sPE (*n* = 8) or nPTB (*n* = 8). White arrows indicate PMNs. Scale bar = 75 μm. **c** Immunohistochemical staining for Neutrophil Elastase in placental sections from patients with sPE (*n* = 8) or nPTB (*n* = 8). Black arrows indicate PMNs. Scale bar = 50 μm. Error bars, mean ± SD. The data were analyzed by an unpaired *t* test. *P < 0.05; **P < 0.01

Circulating maternal immune cells, especially PMNs, which are the most abundant cells, and STBs form the second immune interface between the mother and fetus and involve systemic immune responses^7^. Thus, the number and percentage of PMNs were retrospectively evaluated in the peripheral blood of women with sPE (*n* = 37) and normal pregnant women of matched gestational age (*n* = 46) from their routine blood examination results (Table S2). We found that both the number and percentage of PMNs were significantly increased in the peripheral blood of sPE patients (Fig. 2a). Moreover, PMNs in the human placenta were detected by immunostaining with an anti-CD66b antibody and anti-neutrophil elastase antibody. The results showed that PMNs were located in the villous core and intervillous space or adjacent to the STBs and that the number of PMNs was also significantly increased in the placenta of sPE patients (Fig. 2b, c).

### IL-32β induces the activation of PMNs

**Fig. 3:**
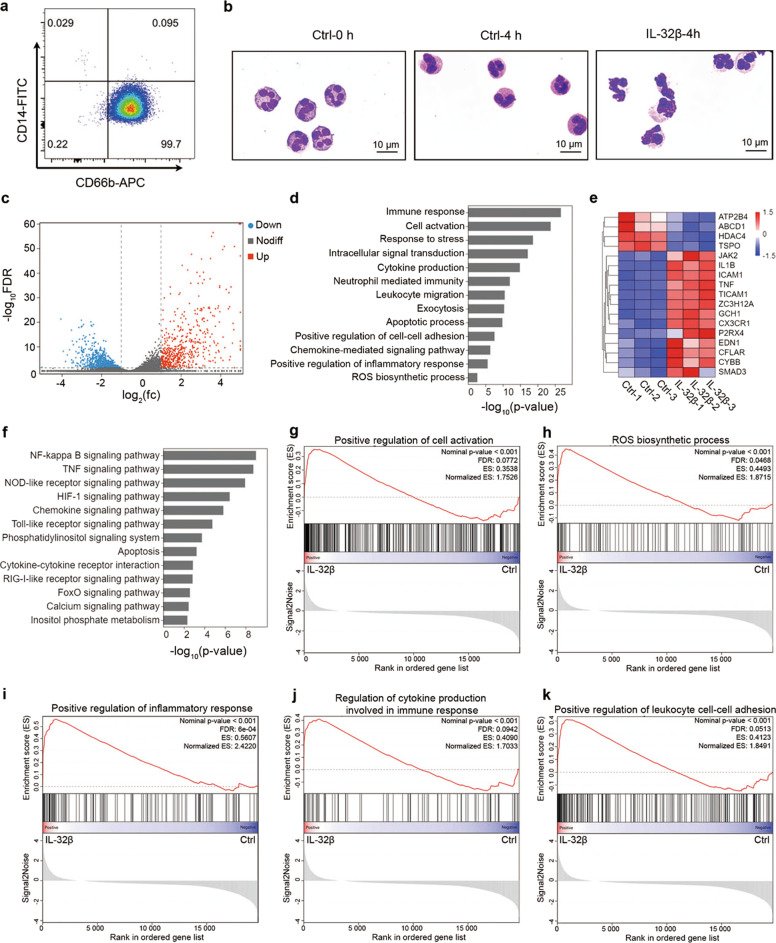
Transcriptome profiling reveals that IL-32 induces the activation of PMNs. **a** Flow cytometry dot plot of freshly isolated PMNs from normal pregnant women. **b** Wright-Giemsa staining of freshly isolated PMNs and PMNs treated with or without 100 ng·mL^−1^ IL-32β for 4 h. Scale bar = 10 μm. **c** Volcano plot representing DEGs in PMNs treated with 100 ng·mL^−1^ IL-32β for 4 h versus control PMNs. Blue dots represent downregulated DEGs, and red dots represent upregulated DEGs (FDR < 0.05 and fold change ≥2). **d** Major enriched GO biological processes of the DEGs. **e** Clustering of DEGs within the “ROS biosynthetic process” term. **f** Major enriched KEGG pathways of the DEGs. **g-k** The GSEA results of “Positive regulation of cell activation” (**g**), “ROS biosynthetic process” (**h**), “Positive regulation of inflammatory response” (**i**), “Regulat**i**on of cytokine production involved in immune response” (**j**), and “Positive regulation of leukocyte cell-cell adhesion” (**k**).

To test the effect of IL-32β on PMNs, PMNs were purified from the peripheral blood of normal pregnant women. The PMN purity was >98%, as determined by both FACS analysis (Fig. 3a) and Wright-Giemsa staining (Fig. 3b). After four hours of culture in RPMI 1640 medium containing 10% heat-inactivated FBS, PMNs without any treatment exhibited a spherical shape and smaller size, while PMNs treated with 100 ng·mL^−1^ IL-32β exhibited irregular shapes (Fig. 3b), which suggests that IL-32β activates PMNs. Therefore, RNA was extracted from three paired PMN samples treated with IL-32β or PBS 4 h after transfection and subjected to transcriptome analysis. As shown in the volcano plot, a total of 1442 DEGs were identified after IL-32β treatment (FDR <0.05 and fold change ≥2), among which 550 genes were upregulated and 892 genes were downregulated (Fig. 3c).

GO enrichment analysis was performed to assess the DEGs. The major 13 enriched GO biological processes were related to the immune response, cell activation, cytokine production, the apoptotic process, regulation of cell migration and adhesion, and the ROS biosynthetic process (Fig. 3d). As shown in Fig. 3e, the term “ROS biosynthetic process” was significantly associated with the upregulated genes. Next, we classified the DEGs using KEGG analysis, and 13 pathways identified as highly enriched are listed in Fig. 3f. Furthermore, using GSEA, we identified that biological processes such as “Positive regulation of cell activation” (Fig. 3g), “ROS biosynthetic process” (Fig. 3h), “Positive regulation of inflammatory response” (Fig. 3i), “Regulation of cytokine production involved in immune response” (Fig. 3j) and “Positive regulation of leukocyte cell-cell adhesion” (Fig. 3k) were positively regulated in PMNs treated with IL-32β.

**Fig. 4:**
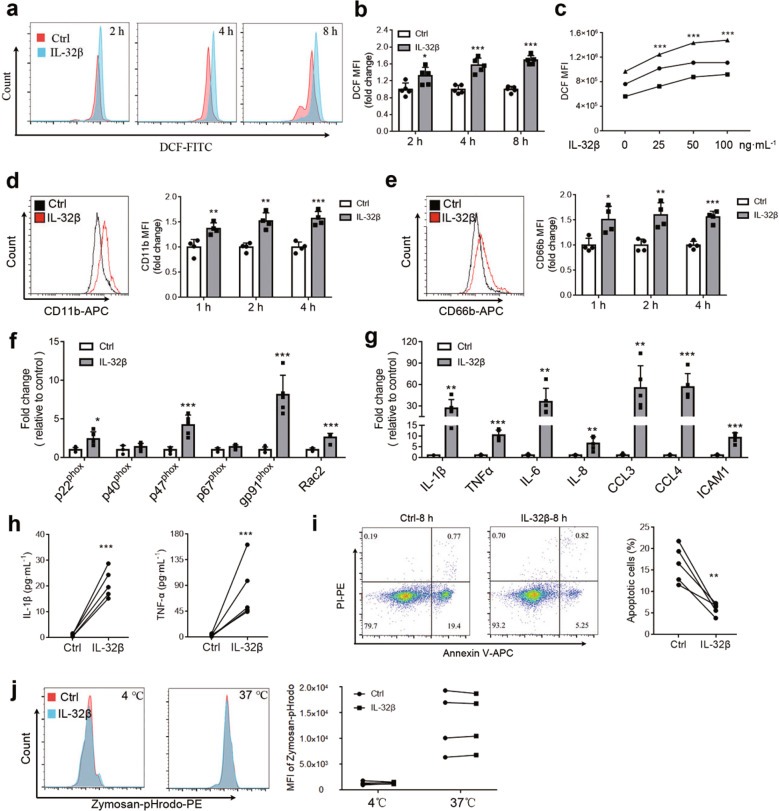
IL-32β induces the activation of PMNs. **a**, **b** Freshly isolated PMNs were treated with 100 ng·mL^−1^ IL-32β for 2, 4, or 8 h. The mean fluorescence intensity (MFI) of DCF was measured by flow cytometry to evaluate ROS production. **c** Freshly isolated PMNs were treated with 0, 25, 50, or 100 ng·mL^−1^ IL-32β for 4 h. ROS production is presented as the MFI of DCF. The expression of CD11b (**d**) and CD66b (**e**) on PMNs treated with 100 ng·mL^−1^ IL-32β for 1, 2 or 4 h. **f** q-PCR analysis of genes involved in NOX in PMNs treated with 100 ng·mL^−1^ IL-32β for 4 h. **g** q-PCR analysis of cytokine genes in PMNs treated with 100 ng·mL^−1^ IL-32β for 4 h. **h** ELISA analysis of IL-1β and TNFα in the culture supernatants of PMNs treated with 100 ng·mL^−1^ IL-32β for 8 h. **i** The apoptosis of PMNs treated with or without 100 ng·mL^−1^ IL-32β for 8 h was analyzed by flow cytometry. **j** The phagocytic activity of PMNs pretreated with or without 100 ng·mL^−1^ IL-32β for 4 h was detected using pHrodo zymosan and analyzed by flow cytometry. The presented results are from at least three independent experiments. The data in (**b**) and (**d–g**) were analyzed by an unpaired *t* test, the data in (**c**) were analyzed by two-way ANOVA with Tukey’s multiple comparisons test, and the data in (**h**), (**j**) were analyzed with a paired *t* test. Error bars, mean + SD. *P < 0.05; **P < 0.01; ***P < 0.001

To verify the effects of IL-32β on PMNs, we next examined PMN ROS production, activation markers and cytokine production after IL-32β treatment. Intracellular ROS levels in PMNs were detected with DCFH-DA (Fig. 4a). As shown in Fig. 4b, 100 ng·mL^−1^ IL-32β treatment significantly induced ROS production in PMNs, especially after 4 h and 8 h (Fig. 4b). Moreover, the IL-32β concentration of 100 ng·mL^−1^ was more effective than that 25 or 50 ng·mL^−1^ (Fig. 4c) after treatment for 4 h. CD11b and CD66b are markers of PMN activation that are rapidly expressed on the cell surface after stimulation^13,35^. Compared with control treatment, IL-32β significantly induced the surface expression of CD11b (Fig. 4d) and CD66b on PMNs (Fig. 4e).

ROS production in PMNs is dependent on the formation of the NOX complex, which contains gp91^phox^ (CYBB), p22^phox^ (CYBA), p47^phox^ (NCF1), p67^phox^ (NCF2), p40^phox^ (NCF4) and Rac2 (RAC2)^36,37^. Thus, we evaluated the expression of multiple  NOX subunits using q-PCR (Fig. 4f) and found that the mRNA levels of p22^phox^, p47^phox^, gp91^phox^ and Rac2 were dramatically increased after IL-32β treatment. Moreover, increased mRNA expression levels of IL-1β, TNFα, IL-6, IL-8, CCL3, CCL4, and ICAM-1 (Fig. 4g) were also observed in PMNs treated with IL-32β. We then examined the levels of IL-1β and TNFα in the culture supernatant of PMNs and found that they were dramatically upregulated by IL-32β (Fig. 4h). In addition, IL-32β decreased PMN apoptosis (Fig. 4i) but did not affect the phagocytotic activity of PMNs in vitro (Fig. 4j). These results indicate that IL-32β can activate PMNs in vitro.

### IL-32β-pretreated PMNs promote the activation of vascular endothelial cells

**Fig. 5:**
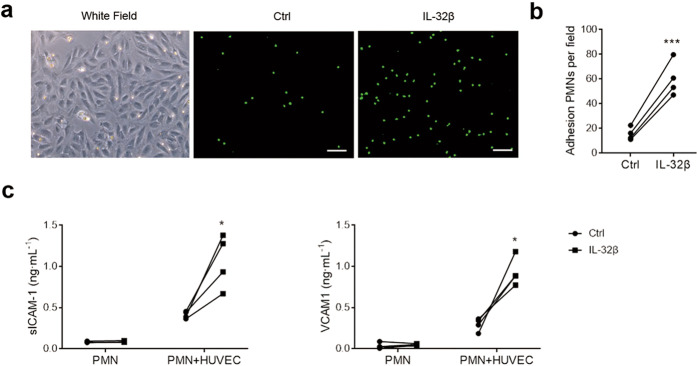
IL-32β pretreatment enhances PMN adhesion to and activation of HUVECs. **a** Representative images of PMNs adhered to HUVEC monolayers. Scale bar = 100 μm. PMNs labeled with calcein-AM and pretreated with or without 100 ng·mL^−1^ IL-32β (IL-32β was removed by washing with PBS twice) were applied to HUVECs and cultured for 1 h. Any nonadherent cells were removed by gently washing the wells twice with PBS. The adhesion of the PMNs to the HUVECs was evaluated by counting the residual cell numbers under a microscope (**b**). **c**, The levels of sICAM-1 and VCAM-1 in PMN culture supernatants and PMN-HUVEC culture supernatants. PMNs were pretreated with or without 100 ng·mL^−1^ IL-32β for 1 h. Then, they were applied to HUVECs and cocultured for 24 h or plated in 24-well plates and cultured for 24 h. The culture supernatants were collected for ELISA. The results are from four independent experiments. The data were analyzed by a paired *t* test. *P < 0.05; ***P < 0.001

The activation of endothelial cells is considered a key pathological event in the second stage of PE [2]. To test the effect of IL-32β-activated PMNs on endothelial cells, primary HUVECs were isolated and cocultured with PMNs given different pretreatments. Calcein-AM-labeled PMNs were pretreated with IL-32β or PBS for 1 h and then applied to a monolayer of HUVECs. One hour later, any nonadherent cells were removed by gentle washing, and the adherent PMNs were evaluated under a microscope. The results showed that IL-32β pretreatment increased the adhesion of PMNs to HUVECs (Fig. 5a, b). The levels of sICAM-1 and VCAM-1 were detected in the supernatant after PMNs treated with IL-32β were cocultured with HUVECs for 24 h. The results showed that the levels of sICAM-1 and VCAM-1, markers of endothelial cell activation, were significantly increased (Fig. 5c).

### IL-32β-induced PMN activation is dependent on ROS

**Fig. 6:**
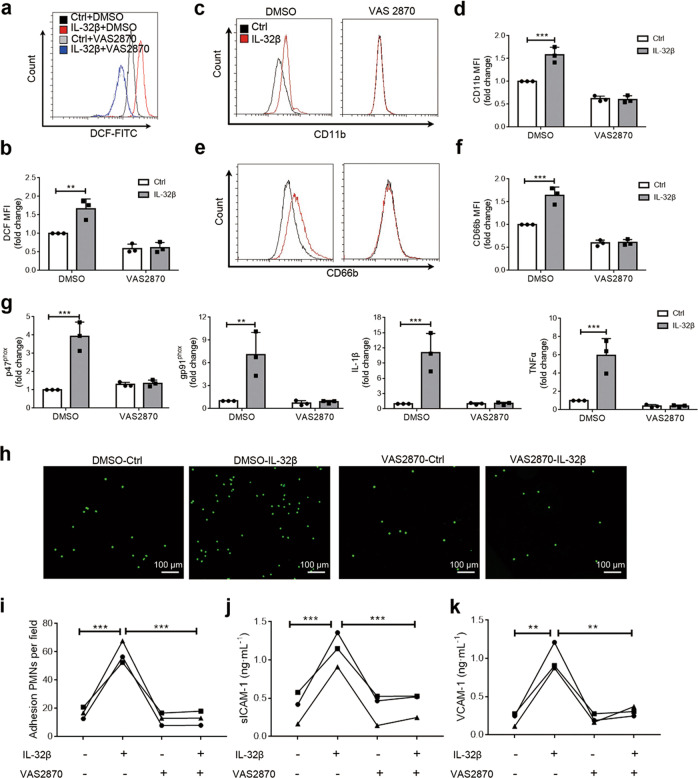
IL-32β induces the activation of PMNs in a ROS-dependent manner. Freshly isolated PMNs were preincubated with DMSO or 10 μM VAS 2870, an NOX inhibitor, for 30 min before treatment with 100 ng·mL^−1^ IL-32β. Four hours later, the production of ROS was detected using DCFH-DA (**a**, **b**), and the expression levels of CD11b (**c**, **d**) and CD66b (**e**, **f**) on the surface of PMNs were detected by flow cytometry. VAS 2870 inhibited the production of ROS (**a**, **b)** and surface expression of CD11b (**c**, **d**) and CD66b (**e**, **f**) induced by IL-32β. **g**, VAS 2870 inhibited the expression of gp91^phox^, p47^phox^, TNFα and IL-1β, which was detected by q-PCR. **h–i**, VAS 2870 inhibited the IL-32β-induced adhesion of PMNs to HUVECs. PMNs given different pretreatments were collected and applied to HUVECs for 1 h. Scale bar = 100 μm. **j**, **k**, The levels of sICAM-1 and VCAM-1 in PMN-HUVEC coculture supernatants. PMNs given different pretreatments were applied to HUVECs and cocultured for 24 h. The results are from at least three independent experiments. The data were analyzed by two-way ANOVA with Sidak’s multiple comparisons test. Error bars, mean + SD. **P < 0.01; ***P < 0.001

ROS production in PMNs is dependent on the formation of the NOX complex^37,38^. To understand the role of ROS in the IL-32β-induced activation of PMNs, VAS 2870, an NOX inhibitor, was added 30 min before IL-32β treatment. We found that VAS 2870 inhibited the production of ROS (Fig. 6a–b) and surface expression of CD11b (Fig. 6c, d) and CD66b (Fig. 6e, f) induced by IL-32β. VAS 2870 also suppressed the IL-32β-induced expression of gp91^phox^, p47^phox^, TNFα and IL-1β (Fig. 6g). These results indicated that IL-32β activated PMNs by inducing ROS production. Furthermore, VAS 2870 preincubation inhibited the IL-32β-induced adhesion of PMNs to HUVECs (Fig. 6h, i) and expression of sICAM-1 and VCAM-1 (Fig. 6j, k). Overall, the NOX inhibitor could inhibit the IL-32β-induced activation of PMNs.

### IL-32β activates PMNs mainly by binding to PR3

**Fig. 7:**
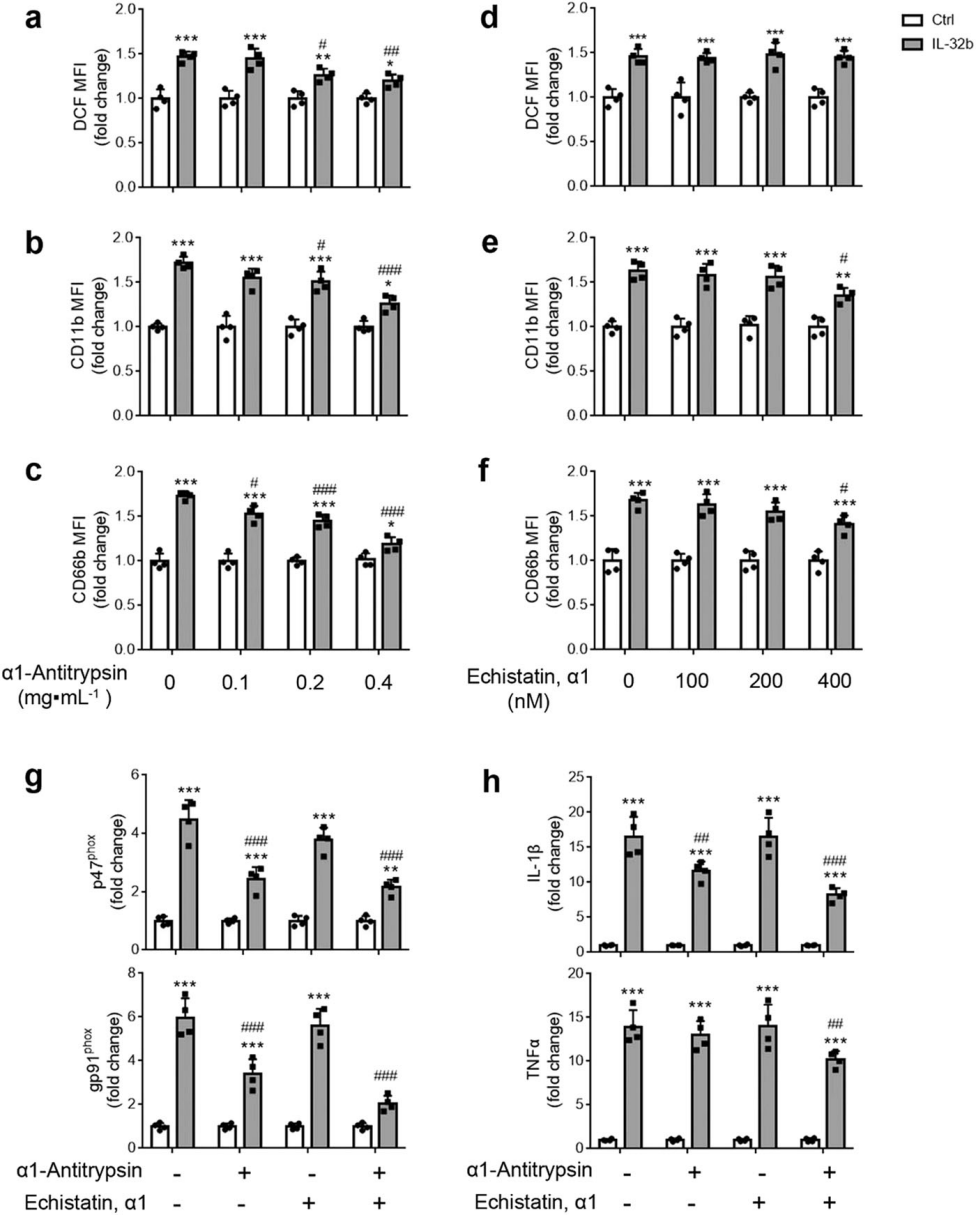
IL-32β activates PMNs mainly by binding to PR3. The PR3 inhibitor α1-antitrypsin inhibited the IL-32β-induced production of ROS (**a**) and surface expression of CD11b (**b**) and CD66b (**c**) in PMNs in a dose-dependent manner. **d** The α_V_β_3_ integrin inhibitor Echistatin, α1 isoform did not inhibit IL-32β-induced ROS production. A high concentration of Echistatin, α1 isoform slightly inhibited the IL-32β-induced surface expression of CD11b (**e**) and CD66b (**f**) on PMNs. **g** The expression levels of gp91^phox^, p47^phox^, TNFα, and IL-1β in PMNs pretreated with 400 nM Echistatin, α1 isoform or 0.4 mg·mL^−1^ α1-antitrypsin. The results are from four independent experiments. The data were analyzed by two-way ANOVA with Tukey’s multiple comparisons test. Error bars, mean + SD. *P < 0.05; **P < 0.01; ***P < 0.001, IL-32β compared with the corresponding Ctrl. ^#^*P* < 0.05; ^##^*P* < 0.01; ^###^*P* < 0.001 versus vehicle in the IL-32β-treated group

To clarify whether IL-32β binds to PR3^26,27^ or the integrin α_V_β_3_^28,29^ on PMNs, freshly isolated PMNs were preincubated with α-1 antitrypsin, which is a naturally occurring serine protease inhibitor^26,27^, or Echistatin, α1 isoform, which is an integrin α_V_β_3_ inhibitor, for 30 min before treatment with 100 ng·mL^−1^ IL-32β. We found that α-1 antitrypsin could inhibit the IL-32β-induced production of ROS (Fig. 7a) and surface expression of CD11b (Fig. 7b) and CD66b (Fig. 7c) in PMNs. A high concentration of Echistatin, α1 isoform could only slightly inhibit the surface expression of CD11b and CD66b on PMNs and did not inhibit ROS production (Fig. 7d–f). Moreover, α-1 antitrypsin inhibited the IL-32β-induced expression of gp91^phox^, P47^phox^ and IL-1β in PMNs, and Echistatin, α1 isoform enhanced the inhibitory effect of α-1 antitrypsin. These results indicate that IL-32β activates PMNs mainly through PR3.

### IL-32β administration induces a PE-like phenotype in pregnant mice

**Fig. 8:**
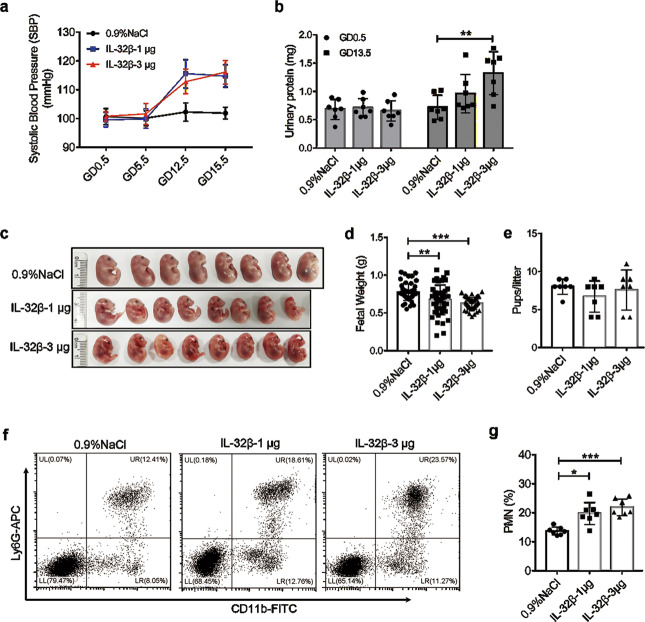
IL-32β administration leads to a PE-like phenotype in pregnant mice. The SBP (**a**) and total urinary protein ((**b**)) of pregnant mice treated with saline (*n* = 7), 1 µg of IL-32β (*n* = 7) or 3 µg of IL-32β (*n* = 7). **c** Representative pictures of fetuses on GD17.5. **d** The weights of fetuses on GD17.5 in the different groups treated with saline (*n* = 56), 1 µg of IL-32β (*n* = 47) or 3 µg of IL-32β (*n* = 52). **e** The number of pups per litter. **f**, **g** The percentage of CD11b^+^Ly6G^+^ PMNs in the peripheral blood was detected by flow cytometry on GD17.5. Data were analyzed by one-way ANOVA with Tukey’s multiple comparisons test. Error bars, mean ± SD. *P < 0.05; **P < 0.01; ***P < 0.001

To test our hypothesis that IL-32β activates PMNs and participates in the development of PE, we established a mouse model. Twenty-one pregnant mice were randomly divided into three groups (intravenous injection of saline *n* = 7, IL-32β-1 μg *n* = 7 and IL-32β-3 μg *n* = 7; injections on GD8.5, GD11.5, GD14.5, and GD16.5). As shown in Fig. 8a, the systolic blood pressure (SBP) of the mice in these three groups was similar (*p* > 0.05) on GD0.5 and GD5.5. After IL-32β administration, regardless of whether 1 μg or 3 μg was administered, the SBP of the mice treated with IL-32β exhibited significant elevations on GD12.5 and GD15.5 compared to that of the saline-treated mice (Fig. 8a). Moreover, elevated total urinary protein levels were observed on GD13.5 in the mice treated with 3 µg of IL-32β (Fig. 8b, saline vs. IL-32β-3 μg, 0.7188 ± 0.08106 vs. 1.324 ± 0.1432 mg). The weight of fetuses from mice treated with 1 μg or 3 μg of IL-32β was decreased on GD17.5 (Fig. 8c, d), although the number of pups per litter was not significantly different among the three groups (Fig. 8e). Furthermore, the percentage of PMNs (CD11b^+^Ly6G^+^) in the peripheral blood was increased in both the IL-32β-1 μg and IL-32β-3 μg groups on GD17.5 (Fig. 8f, g). Taken together, the above results indicated that IL-32β administration led to a PE-like phenotype.

## Discussion

IL-32 has been recognized as a new inflammatory cytokine and a strong inducer of other proinflammatory cytokines, including TNF-α, IL-1β, IL-6, and IL-8^18–20^. In this study, we showed that IL-32 was highly expressed in the first-TM placenta and that this expression progressively decreased as pregnancy advanced, which was in accordance with hypothesized proinflammatory environment required for embryo implantation and the anti-inflammatory environment required for the second trimester^39^. However, in sPE patients, IL-32β expression was upregulated in placental tissues and serum in the third trimester without labor. It has been reported that IL-32β promotes vascular inflammation^20^ and neuroinflammation^21^. IL-32β is associated with increased cancer cell death, invasion and migration and plays an important role in regulating the antitumor immune response, which also influences tumor progression^22,23^. This study showed that the increased numbers of PMNs in the placenta came into contact with the upregulated IL-32 on the STBs of PE patients, providing the chance to activate the PMNs.

PMNs are involved in various stages of pregnancy, and their functions are complicated. Activated PMNs promote implantation and assist with the process of parturition. However, aberrant or overt activation of PMNs plays a key role in the development of PE^9–11^. Recently, Amsalem et al. identified a novel PMN subset with a physiological, angiogenic role in the second-trimester decidua^40^. Nadkarni et al. demonstrated that a PMN population generated by exposure to pregnancy hormones was able to induce a unique population of T cells that had regulatory-like and proangiogenic phenotypes in pregnancy^41^. In this study, we found that PMN levels were increased in PE patients and that IL-32β could activate PMNs in vitro. Moreover, using a pregnant mouse model, we found that intravenous injection of IL-32β could induce a PE-like phenotype and increase the number of PMNs. However, the function of PMNs in spiral artery development in the maternal decidua needs to be explored in future work.

ROS are highly reactive free radicals that can cause cellular damage by inducing lipid peroxidation, protein and amino acid modifications, and DNA oxidation^37,38^. In PE, maternal circulating and placental tissue levels of ROS and lipid peroxides are increased, whereas those of antioxidants are decreased^42–44^, resulting in oxidative stress. Excess ROS damage the placenta and thereby contribute to the activation of the maternal immune system, which is believed to be a mediator of maternal endothelial dysfunction^45^. Not only the placenta but also maternal leukocytes and the maternal endothelium are sources of free radical synthesis^45,46^. PMNs in the peripheral circulation^9–11^ and placental tissue^47–49^ of patients with PE are known to be activated and produce elevated amounts of ROS. In this study, we found that IL-32β, which is highly expressed in trophoblast cells, induced the production of ROS in PMNs through NOX and that these activated PMNs played an important role in disrupting the function of the endothelium. It has been reported that treatment with IL-32 (0.5-1 μg·mL^−1^) increases ROS production in cultured primary rat astrocytes^50^, but the underlying mechanism is not clear. In our study, we found that IL-32β induced the expression of NOX subunits, especially gp91^phox^ and p47^phox^. However, IL-32α did not have these effects on PMNs (data not shown).

The specific receptor for IL-32 that facilitates extracellular signaling has not yet been identified. PR3, which exists in soluble and membrane-bound forms, is an IL-32-binding protein^26,27^. IL-32α has been shown to bind to the cell-surface integrins α_V_β_3_ and α_V_β_6_ via the RGD motif. IL-32β can also bind to α_V_β_3_, but it is not affected by the RGD motif^28,29^. Using inhibitors of PR3 and α_V_β_3_, we found that PR3 might be the main binding protein of IL-32β on PMNs. However, we did not rule out the possibility that other receptors for IL-32β exist on PMNs, which requires further study.

In this study, IL-32β pretreatment increased the adhesion of PMNs to endothelial cells and the expression of ICAM-1 and VCAM-1 in HUVECs, indicating that IL-32β-pretreated PMNs promoted the activation of endothelial cells in vitro. Kobayashi et al. reported that IL-32β was the major isoform in endothelial cells and that high expression of IL-32β in HUVECs increased the expression of vascular cell adhesion molecules and subsequent leukocyte adhesion. Importantly, endothelial/hematopoietic expression of IL-32β in transgenic mice elevates inflammation and worsens sepsis^20,51^. However, our results showed that IL-32β not only directly impaired the endothelium (data not shown) but also activated PMNs, which indicated that the effects of IL-32β on endothelial cells were extensive.

In conclusion, our study shows that elevated placental expression of IL-32β participates in the development of PE by activating PMNs and damaging systemic endothelial cells. This study provides new insights into the pathogenesis of PE.

## Supplementary information

Figure S1
