# Supplementary figures and images for: Placenta-derived IL-32β activates neutrophils to promote preeclampsia development
Source: Cell Mol Immunol. 2021 Mar 11;18(4):979–91. doi: 10.1038/s41423-021-00636-5 (PMC8115232; doi:10.1038/s41423-021-00636-5)

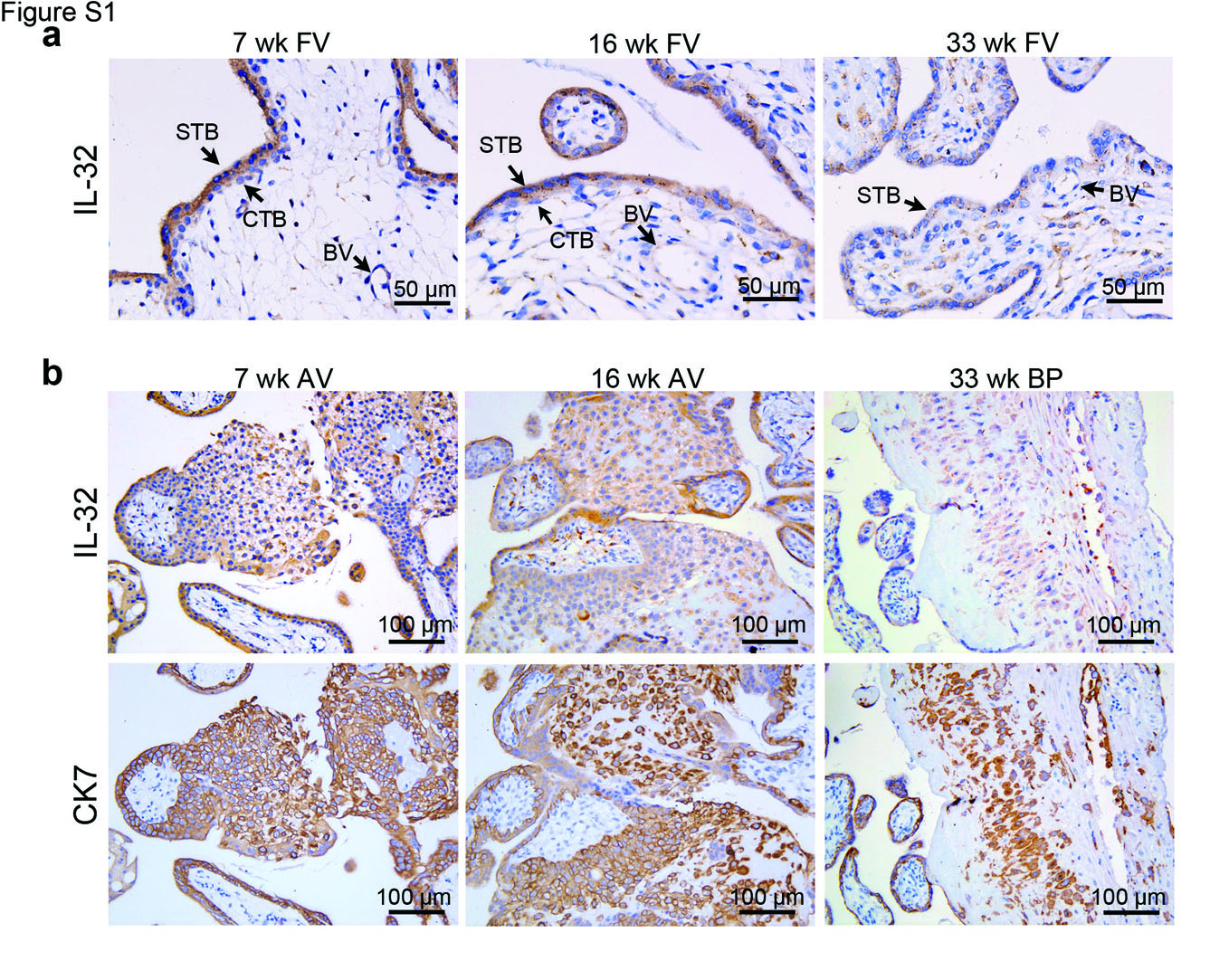

Supplement: Supplementary file 1 — Figure S1 [file 41423_2021_636_MOESM1_ESM.jpg]
